# Surface Acoustic Wave (SAW) for Chemical Sensing Applications of Recognition Layers †

**DOI:** 10.3390/s17122716

**Published:** 2017-11-24

**Authors:** Adnan Mujahid, Franz L. Dickert

**Affiliations:** 1 Department of Analytical Chemistry, University of Vienna, Währinger Straße 38, A-1090 Vienna, Austria; adnanmujahid.chem@pu.edu.pk; 2 Institute of Chemistry, University of the Punjab, Quaid-i-Azam Campus, Lahore 54590, Pakistan

**Keywords:** surface acoustic wave (SAW), shear-horizontal surface acoustic wave (SH-SAW), shear transverse wave (STW), RFID-tags, piezoelectric, chemical sensors, recognition layers

## Abstract

Surface acoustic wave (SAW) resonators represent some of the most prominent acoustic devices for chemical sensing applications. As their frequency ranges from several hundred MHz to GHz, therefore they can record remarkably diminutive frequency shifts resulting from exceptionally small mass loadings. Their miniaturized design, high thermal stability and possibility of wireless integration make these devices highly competitive. Owing to these special characteristics, they are widely accepted as smart transducers that can be combined with a variety of recognition layers based on host-guest interactions, metal oxide coatings, carbon nanotubes, graphene sheets, functional polymers and biological receptors. As a result of this, there is a broad spectrum of SAW sensors, i.e., having sensing applications ranging from small gas molecules to large bio-analytes or even whole cell structures. This review shall cover from the fundamentals to modern design developments in SAW devices with respect to interfacial receptor coatings for exemplary sensor applications. The related problems and their possible solutions shall also be covered, with a focus on emerging trends and future opportunities for making SAW as established sensing technology.

## 1. Introduction

Modern chemical sensor designs focus on fast and highly responsive transducer devices that can readily transform chemical responses into measurable electronic signals. These devices should be robust and stable under all working conditions, especially in multifaceted environments. This includes their ability to generate and transform sensor signals in thermally and chemically corrosive mixtures. Moreover, their ability for wireless communication [1,2] and small size should make their integration with remote devices possible [3] for developing portable and miniaturized detection chips. The accuracy of such sensors should be comparable to traditional analytical instruments so that they can compete and provide real alternates. There are stringent yet important features of an ideal chemical sensor that must be met for commercially acceptable sensing tools for a broad spectrum of uses. There are various types of transducer devices, such as acoustic [4], electrochemical [5], optical [6], magnetic [7,8] and thermo-responsive [9,10] which have been comprehensively studied for chemical sensor research and development.

Different from all other types of devices, acoustic resonators are considered as universal transducers because mass is a primary feature of any target and even if it does not possess any electric/magnetic, optical and thermal properties, it still can be detected by acoustic sensors down to levels as low as picograms [11]. This feature makes acoustic/gravimetric transducers unique in the sensing domain and due this reason they are widely accepted for developing label-free sensors. The principle of acoustic devices relies on the *piezoelectric effect* whereby a certain material under stress becomes polarization leading to the generation of a potential difference. The reverse is also true, so when a voltage is applied to such materials, they undergo deformation causing a *converse piezoelectric effect*. The phenomena was noted by the Curie brothers [12,13] and later mathematically verified by Lipmann [14]. Typical examples of piezoelectric materials are quartz, Rochelle salt, lithium niobate and lithium tantalate, gallium arsenide and gallium orthophosphate and others. Piezoelectric materials have found numerous applications in many fields such as power generators, frequency standards, microelectronics, biomedical imaging and sensing systems. Due to their excellent actuating features, they have been widely used in developing smart sensing devices.

Surface acoustic wave (SAW) devices are among some of the first generation acoustic resonators that were first discussed about four decades ago [15,16]. In 1974, Piezo Technology (Orlando, FL, USA) and Texas Instruments (Dallas, TX, USA) jointly published an article [17] on ultra-high frequency SAW resonators in which they were able to work on 400 MHz devices. With some recommendations, they also predicted that SAW devices would undergo considerable progress in coming years. Since then, SAW resonators have been found to be highly valuable in diverse fields, including frequency filters for electronic devices including wireless communication tools and especially for developing smart miniaturized sensors. Their exceptionally high frequency among acoustic devices, small size, faster response, high ruggedness, and integration ability with different receptor materials are some of the highlighted features of SAW devices. These characteristics make them highly suitable in the sensing field for a variety of targets such as volatile organic compounds (VOCs), toxic gases, chemical warfare agents, explosives, and bio-analytes including proteins, yeast cells for biotechnological process monitoring and cancer cells for clinical diagnostics. For developing such sensors, there is an extensive range of receptor interfaces, for example supramolecular coatings [18], metal oxide nanostructures [19], carbon nanotubes and composites [20,21], self-assembled monolayers [22], molecularly imprinted polymers [23] and natural receptors [24] that are reported for potential chemical sensing applications. Nonetheless, typically the selection of a particular receptor mainly depends upon its straightforward synthesis and integration with SAW, highly sensitivity and selective binding with the target, regeneration for reusing the sensing surface and being able to keep coatings intact and stable without losing their recognition properties.

Apart from coating materials, the performance of SAW resonators for sensing are associated with many variables like the nature of the piezoelectric substrate, damping problems in the liquid phase, temperature effect, frequency dependency and design fabrication for enhanced response. These are important and testing parameters for making SAW a competitive chemical sensor transducer. In this article, we shall describe imperative design and operational details of SAW for sensing perspectives, including related challenges. Furthermore, their significant contributions for both gas [25,26,27] and liquid phase sensing [28,29,30] would be reviewed with a view to potential receptors coatings for typical applications. The use of microfluidics, RFID-tags with recognition layers and commercial systems using gas chromatography and SAW combinations are also highlighted. Finally, the challenges related to transducer design and recognition layers are also described, with a focus on establishing competitive SAW sensors and identifying future opportunities.

## 2. SAW Overview

### 2.1. SAW Design and Operation

**Figure 1:**
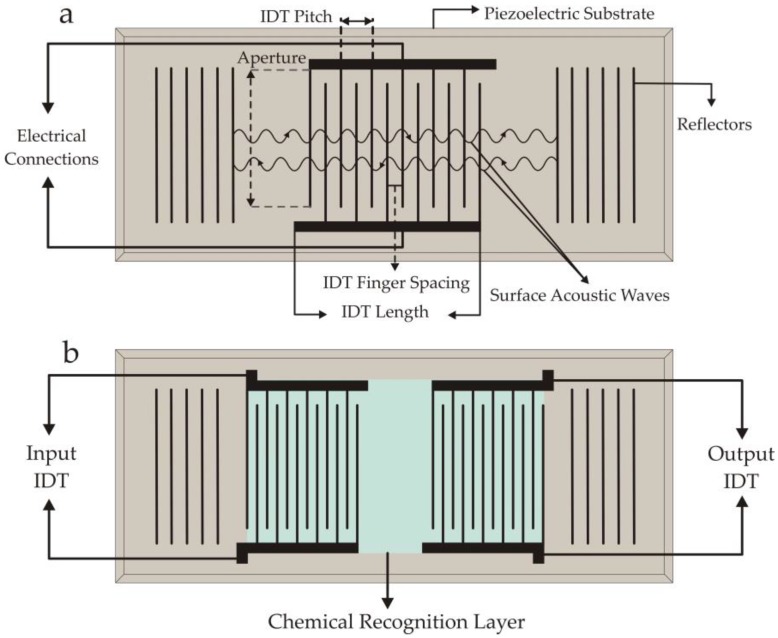
(**a**) A typical design of a one port SAW resonator; (**b**) a two port SAW device having a chemical recognition layer.

SAW devices are made of piezoelectric materials on which a periodic comb-shaped interdigital transducer (IDT) pattern is developed by a photolithographic or other process. These electrodes are generally made of inert metals or alloys, for example Au, Cr/Au/Cr, etc. When an AC voltage is applied to these IDTs, an acoustic wave is generated which travels across the crystal surface, however, perpendicular to the IDTs but in an away direction. The velocity of surface waves is 10^−5^ times that of light waves. These acoustic waves are confined to the substrate surface, having penetration depths of a few wavelengths which indicates that they possess high surface energy. Lord Raleigh was the first to explain the propagation of waves along the plane surface of elastic solids in his classical paper [31]. A typical SAW resonator is shown in Figure 1a. In this configuration there are specially designed grating reflectors having a period of λ/2 which are used to reflect back the surface waves towards the IDTs for resonance. A single port SAW device contains only one set of IDTs, but two reflectors on each side of the crystal for obtaining suitable resonance signals. Single port SAWs are usually considered for making oscillating circuits whereas two port devices are useful for developing special frequency filters.

**Figure 2:**
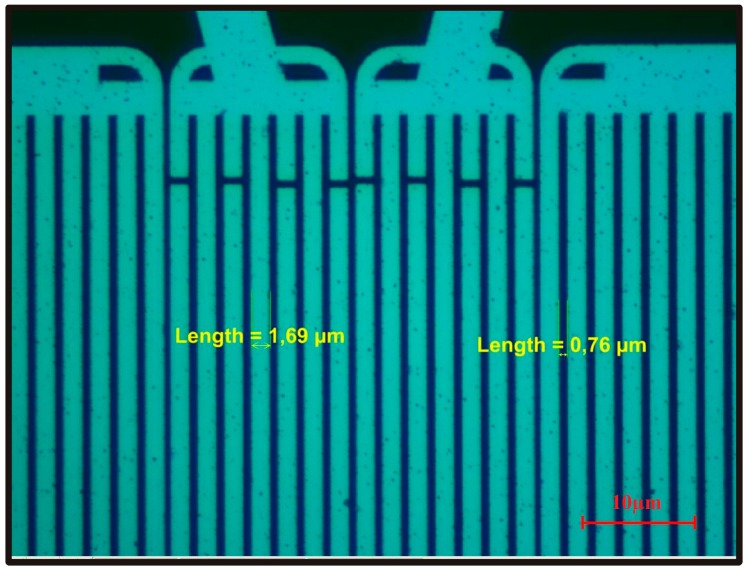
Optical microscope image of two port SAW device showing the IDT electrode width, i.e., 1.69 μm and spacing, i.e., 0.76 μm between two consecutive fingers.

The other type of SAW device fabricated on piezoelectric material is shown in Figure 1b. The delay line represents the distance between two sets of IDTs where usually a chemical recognition layer is deposited. As there are two sets of electrodes on the substrate, therefore these are also termed two-port devices, i.e., one is the transmitter IDTs which may also be called the input electrodes and other is the receiver IDTs or output electrodes. Upon applying a certain voltage, acoustic waves are generated which travel from the transmitter to receiver end, and since they have high energy, any perturbations in the surface wave propagation (due to the interaction between chemical recognition coatings and an analyte) would alter the signals as received at the output IDTs. On receiving at output IDT signal, these waves are again converted into electrical signals and thus, processed. This is the basic principle that is considered in using a SAW device as a highly efficient transducer for sensing applications. Figure 2 presents the optical microscopic image of a two port SAW device showing the IDT electrode width and spacing between two consecutive fingers. The design of a typical SAW device, including the electrode height, spacing, and aperture has a substantial influence on the properties of the acoustic surface waves. For instance, the width of IDTs and their pattern determines the fundamental resonance frequency of the SAW transducer. However, the selectivity is primarily driven by the nature of chemical recognition layer.

In the coming section, we shall focus on all the critical parameters that have a direct or indirect influence on the performance of SAW devices as sensors. This shall cover the selection of piezoelectric substrates and their cutting angles; temperature dependency, frequency sensitivity relationship, and operation in liquid phase as well.

### 2.2. Piezoelectric Materials and Their Cutting Angles

The selection of a piezoelectric material for designing a SAW device mainly depends on the device application, for instance critical parameters include operation in liquid phase, tolerance to thermal stress, damping losses and others. Quartz, i.e., the purest form of silica, is a natural piezoelectric material which is widely used for making surface as well as bulk acoustic wave resonators. Being a low cost material, straightforward fabrication and easy availability make these devices commercially viable for large scale production. There are different ways to cut quartz crystals to obtain the desired wave propagation features, for example the stable temperature (ST)-cut having Euler angles (0°, 132.75°, 0°) is one of the common types which is used in SAW manufacturing. The ST-cut [32] is sometimes also called a Y-cut (X-propagating). Some more temperature stable cut types been that are reported, e.g., leaky stable temperature (LST)-cut for developing different types of SAW resonators with improved temperature-frequency stability and variable wave propagation. Lithium substrates such as lithium tantalate (LiTaO_3_) and lithium niobate (LiNbO_3_) are other prominent piezoelectric materials that are used for SAW fabrication. LiNbO_3_ is processed in many forms such as Y-Z cut, 64° Y-X and 128° Y-X cuts, whereas LiTaO_3_ is often reported as 36° Y-X. LiTaO_3_ and LiNbO_3_ have higher wave velocities than quartz, but on the other hand they are less robust against temperature shifts. The phosphates of aluminum and gallium are thermally stable piezomaterials having lower sound velocity than typical quartz, LiTaO_3_ and LiNbO_3_ substrates. Aluminum nitride (AlN) [33] is another attractive piezoelectric material that exhibits high wave velocity i.e., in the range of 5700 m/s, which is useful for developing high frequency SAW devices. In Table 1 in the next section, we showed that cutting angle has a direct influence on SAW propagation velocity. In general, the selection of a typical piezoelectric material for SAW chemical sensor fabrication depends on their characteristic wave propagation features in certain environments, i.e., gases or liquids. Quartz, LiTaO_3_ and LiNbO_3_ are the most frequent SAW substrates that are widely used along with chemical recognition layers for sensing applications.

### 2.3. Temperature Dependicies

There are several notable factors that influence wave propagation in SAW and among all of them temperature is a critical one. The frequency of an acoustic device changes as a function of temperature which actually sets the foundation for developing SAW temperature sensors [34]. There have been a number of diverse industrial applications of such sensor systems, for example in high speed/voltage motor engines [35], metallurgical vessels [36] and others. SAW devices are workable at temperature as high as 600 °C as reported in [37], however, with a chemical recognition interface, they can work at room temperature for chemical sensor applications. The quantitative relationship between temperature and resonating frequency may be expressed in Equation (1), i.e., by temperature coefficient of frequency (TCF). From the following equation, it is obvious that the TCF is associated with the fundamental frequency of a device:

(1) $$\mathrm{Temperature}\;\mathrm{Coefficient}\;\mathrm{of}\;\mathrm{Frequency}\;\mathrm{(TCF)}\;=\;\;\frac{1}{f_{o}\;}\;\times \;\frac{df_{o}}{dT}$$

Apart from resonating frequency, TFC values vary depending upon the nature of material and its cutting angle structure. From the Table 1, it can be seen that quartz is the most stable piezoelectric material, whereas LiTaO_3_ and LiNbO_3_ shows frequency drifts under temperature fluctuations. Gallium orthophosphate (GaPO_4_) and langasite (La_3_Ga_5_SiO_14_) possess high thermal stability. Aluminum nitride (AlN) is also a thermally stable piezoelectric material and shows a much higher SAW velocity compared to other piezoelectric materials.

It is obvious that quartz has a zero TCF value showing its ability to remain stable against temperature variations during measurements. For chemical sensor applications frequency shifts due to temperature fluctuations needs to be avoided thus, piezomaterials having low TCF are recommended. Furthermore, the problem of frequency shifts due to temperature variations during measurements can also be solved by integrating a suitable temperature compensation element, i.e., an additional SAW device as reference channel.

### 2.4. Operation in Liquid Mediums

**Figure 3:**
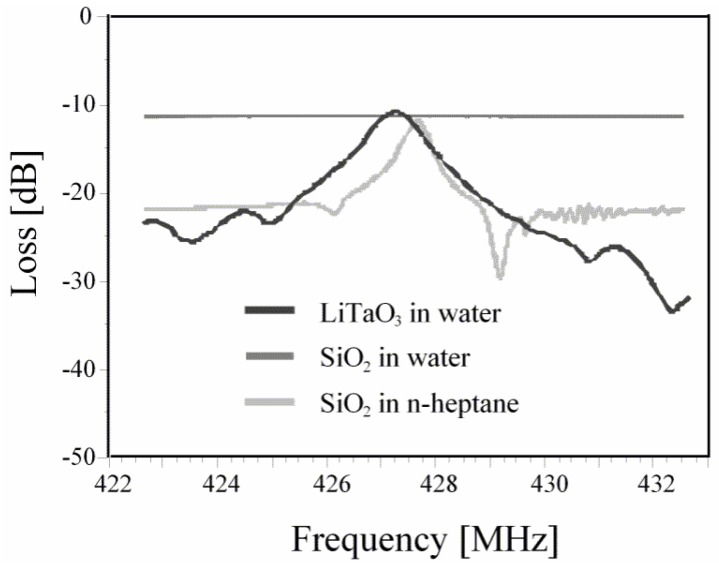
Damping spectra of LiTaO_3_ and SiO_2_ in water. Damping of SiO_2_ in *n*-heptane is also shown. It is seen here that SiO_2_ has no resonance spectrum in water due to its high dielectric constant (≈80). However, SiO_2_ shows some damping spectra in *n*-heptane that has dielectric constant of about 1.9. LiTaO_3_ exhibits suitable resonance in water, demonstrating its ability to work in aqueous phases. Adapted with permission from [45], copyright (2010) Elsevier.

SAW devices having interfacial recognition layers are comprehensively explored for gas phase sensing applications. However, in liquid phase measurements, the longitudinal phase of Rayleigh waves are damped due to viscosity of the medium and this results in a loss of acoustic energy from the surface to the bulk of the liquid. This ultimately limits SAW applications in liquids, although, the problem can be solved by adjusting the cutting angle of the substrate material to produce shear horizontal waves that propagate along the surface of substrate. These devices are called shear horizontal surface acoustic wave (SH-SAW) units [42] or they can be named as horizontally polarized surface shear waves. In this way, acoustic energy loss due to damping can be minimized and thus, the SAW sensing operation can be studied in liquids [43,44]. The other restriction that hampers SAW operation in liquids is the high dielectric constant in the contact medium, e.g., water which has ε ≈ 80. Since the dielectric constant of quartz i.e., the most common SAW substrate material, is about 4, this leads to poor electro-acoustic coupling. In this scenario, piezoelectric materials like LiTaO_3_ which have a dielectric constant value of 43 can be effectively used for sensing in aqueous media. Figure 3 shows the resonance spectrum of quartz (SiO_2_) and LiTaO_3_ in water, where it can be seen that for quartz there is no signal thus, cannot be used in aqueous phase, while for LiTaO_3_ we can observe a suitable damping spectrum [45]. Furthermore, quartz is only able to show some damping spectra in *n*-heptane which has a dielectric constant of about 1.9.

Apart from the substrate material, the design of a surface acoustic resonator is also important for operation in liquids for instance Lamb Wave devices with confined thickness or Love Wave resonators having a wave guiding interfacial layer may also be used for liquid phase sensing. In the case of Lamb Wave devices the thickness of the substrate is in the micrometer range, which makes them mechanically fragile. For Love Wave sensors, the transducer is covered with a guiding surface layer which protects the patterned IDEs on the substrate from highly conductive solvents.

### 2.5. Resonating Frequency and Senstivity Relationship

SAW resonators are well known for their exceptionally high frequency which makes them potentially suitable in mass sensing applications. The mathematical correlation between mass loading and the fundamental resonance frequency of piezoelectric materials was first explained by Sauerbery [46]. This relation explains the frequency shifts as a result of mass loading taking into consideration all the important parameters of the substrate material. Equation (2) shows the frequency shift relationship with the fundamental resonating frequency for a typical SAW device:

(2) $$\Delta f=-\frac{k\Delta mf_{o}^{2}}{A}$$

**Figure 4:**
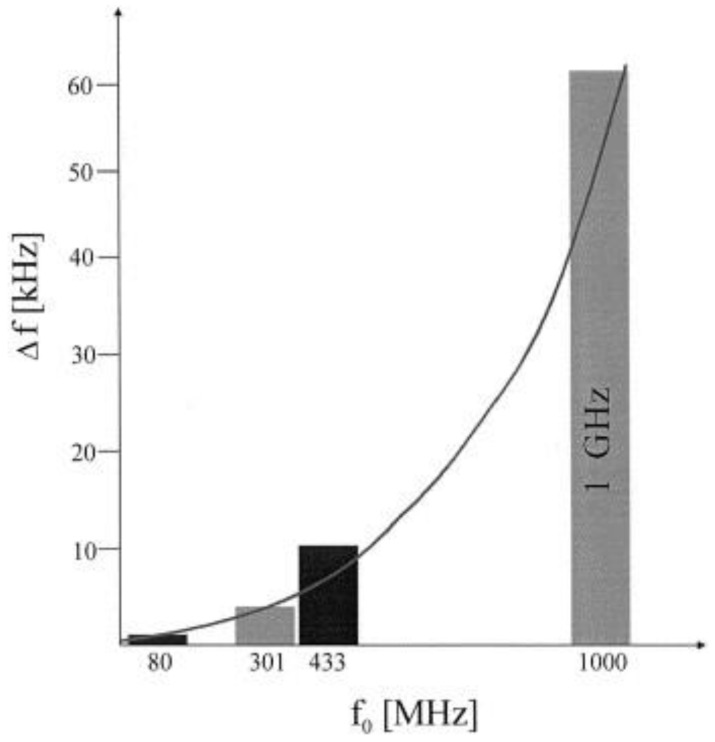
Sensor responses of different SAW devices for 1000 ppm of toluene. All the devices are coated with the same recogntion layer i.e., permethylated β-cyclodextrin linked with hexafluorobenzene having a layer height of 60 nm, and the basic frequencies of the devices were 80, 301, 433 and 1000 MHz. Adapted with permission from [47], copyright (1998) Elsevier.

In this equation, ∆*f* is the frequency shift due to mass loading, *f_o_* is the fundamental resonance frequency, ∆*m* is the loaded mass, *A* is the area and *k* is material constant. This shows that frequency response/shift due to mass loading increases as a function of the increasing fundamental resonance frequency of the device. The increase in frequency shift is parabolic. Unlike bulk acoustic wave devices, SAW resonators can be designed for much higher resonating frequencies i.e., from hundreds of MHz to the GHz range, which is significantly higher than quartz crystal microbalance (QCM). This feature makes SAW devices exceedingly favorable in sensing applications, with superior sensitivity, especially where dealing with trace analyte concentrations. Figure 4 present the sensor responses of four different SAW devices for 1000 ppm of toluene, where all the SAW devices were coated with same recognition layer [47] but having variable resonating frequencies starting at 80 MHz and going to 1 GHz. From this figure, it is clear that by increasing the fundamental frequency, the sensor response increases in a parabolic way, thus showing the experimental evidence for a frequency sensitivity relationship. While combining recognition layers with high frequency SAW resonators for sensing, the thickness of the coating material may be reduced down to monolayers, which results in a shorter response time due to a faster sorption-desorption process. Thus, increase in fundamental resonance frequency leads to enhanced sensitivity and, when combined with thin coatings, to shorter response times.

### 2.6. SAW RFID-Tags

SAW-based radio frequency identification (RFID) [48,49] is a globally recognized system that is used for rapid and automatic tracking/detection applications using a tag and a reader. Here, a remotely placed transmitter/reader sends a radio wave pulse which is received by patterned IDTs on a SAW device and processed into acoustic waves. These waves are passed through a set of reflectors where they produce a unique encoded acoustic wave signature depending upon the pattern and structure of the reflectors. The acoustic waves are sent back to the IDTs where they are converted to radio wave signals and transferred back to the reader. Unlike a typical two-port SAW resonator, RFID ones contain only one IDE port with a unique reflector pattern. SAW-based RFID tags have found numerous applications, ranging from supply chain monitoring to automotive and military applications. In addtion chemical recognition layers can be combined with SAW RFID tags to develop wirelessly integrated chemical sensors. The realization of such SAW devices would be greatly beneficial in remote sensing systems.

## 3. Chemical Recognition Layers

For chemical sensing applications, the SAW interfacial part is covered with a tailored recognition layer that is supposed to interact exclusively with the target molecules. As a result of this interaction, an analyte mass loading takes place on the SAW coating which leads to a drop in the frequency of device, i.e., a sensor response. Thus, a direct relationship between the analyte mass loading and frequency shift is observed. This drop in frequency can be correlated to detect mass shifts as low as a few picograms. The main feature of SAW sensors is their ability to detect mass, which means that if a molecule does not possess notable optical, electrical or any other property, it can still be detected by a gravimetric sensor since mass is a fundamental property of matter. This also suggests that SAW chemical sensors do not require any labelling indicator, unlike other optical or electrochemical sensors.

There is a broad range of receptor materials that can be integrated with SAW as chemical recognition interfaces [50,51,52] for sensing. For instance, this includes supramolecular host-guest structures, metal oxide layers, carbon nanotubes and composites, functional polymeric coatings, biological recognition materials and others. The integration of SAW transducers with such a versatile range of receptors has resulted in diverse sensing applications, e.g., the detection of explosives and chemical warfare agents for military applications, monitoring environmentally toxic and hazardous vapors for air surveillance, label-free detection of microorganisms, including cancer cells, and electronic noses for the analysis of complex mixtures [53,54]. In the coming sections, we shall discuss the potential receptor coatings, along with selected sensing applications in both gas and liquid phases.

### 3.1. Host-Guest Strategies

Supramolecular receptors interact with target molecules according to host-guest chemistry [55,56] which is similar to layer-analyte inclusion. Supramolecalur structures are composed of small molecular units which are assembled to develop a giant structure that offers different ranges of binding interactions for target molecules. The size, shape and functionality of the host cavity structure determines which analytes will be recognized. Therefore, by carefully assembling the smaller units, a larger structure with the desired functionality can be built for specific molecular recognition. A variety of different supramolecular compounds such as cyclodextrins, calixarenes, paracyclophanes, cyclotriphosphazenes and others have been combined with SAWs for toxic organic vapor sensing.

**Figure 5:**
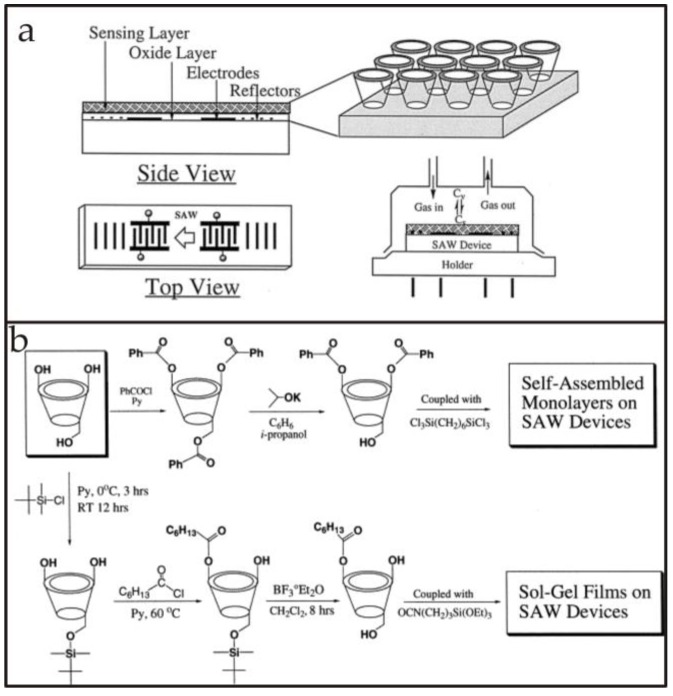
(**a**) Schematic representation of a SAW device with a side view showing the cone shaped cyclodextrin-based monolayer receptors. Furthermore, the device was integrated with a four pin holder which was covered with a special lid having openings for gas inlets and outlets thus, showing the design of measuring cell, whereas (**b**) shows the chemical reactions for synthesizing self-assembled monolayers as well as sol-gel films that were coated on SAW devices. Adapted with permission from [58], copyright (2000) Elsevier.

For instance, the hydrophilic character of β-cyclodextrins (7-membered rings) can be modified by derivatizing the OH groups with alkyl ethers which improves the hydrophobic recognition properties for different analytes and also helps to avoid interferences caused by humidity. Furthermore, cyclodextrins linked with hexafluorobenzene [57] were also used to improve the recognition properties for aromatic analytes and also increase the number of binding sites. Such sensor coatings are highly selective in distinguishing benzene from cyclohexane, for example. This suggests that careful substitution of cyclodextrin terminal groups is important in controlling the hydrophobicity and also shaping the cavities for geometrical fitting of target analytes. Thus, in this way the selectivity offered by host structures is tuned for a range of guest molecules. Li et al. [58] reported cyclodextrin-based self-assembled monolayers and sol-gel-derived multilayers combined with 200 MHz SAWs for organic vapor sensing. The authors proposed that cyclodextrin-monolayers are suitable for studying specific molecular interactions between layer and analyte vapors whereas cyclodextrin sol-gel thick layers are much easier to synthesize with improved responsive character. Figure 5a shows a schematic representation of cyclodextrin derived layers coated on a SAW device for gas sensing. Furthermore, the synthesis schemes of self-assembled monolayers and sol-gel films are also shown in Figure 5b. The relative sensitivity and selectivity of cyclodextrin-derived mono- and multi-layers was compared, which revealed that going from mono- to multi-layer, the relative selectivity remains unchanged, however, the sensitivity increased by a factor of 140. Nonetheless, the thickness of the sensor layer is also important in reducing the noise level during sensor measurement since in another study [59] it was observed that cyclodextrin monolayers result in a homogeneous coating which showed noise of only 4 Hz for a 433 MHz SAW device.

Paracyclophane compounds are a combination of aliphatic and aromatic units which form a cyclic structure. In these host molecules, electronic interactions such as hydrogen bonding, π–π stacking, van der Waals and other electrostatic forces are more dominant than the simple geometrical fitting of guest analytes. Since these structures possess electron donor functionality, it makes them more suitable receptors for electron-deficient analytes. For such recognition layers, the observed sensor effects were correlated with calculated enthalpy values which showed good agreement, especially for distinguishing benzene from toluene, however, some deviations for smaller and polar analytes were also noticed. The introduction of spacer groups of varying length and size between aromatic rings allows the geometrical tailoring of paracyclophanes [60]. The recognition by paracyclophanes is mainly driven by donor-acceptor mechanism, therefore analytes with more polar character and a larger interface with the host structure shows relatively higher sensor responses. Molecular modeling methods [61,62] could also be used to understand the inclusion process and to design smart sensor coatings with pre-determined sensitivity and selectivity patterns.

Calixarenes are another class of supramolecular structure that has been extensively used as SAW sensor coatings for the detection of organic vapors. Compared to previously described macromolecules, calixarenes offer highly steric flexibility which facilitates the accommodation of guest analytes with complementary geometrical adaptation. The hydrophobic character of calixarenes can be increased by methylation or silylation of hydroxyl group which in turn reduces the interference of humidity and also improves the selectivity. For example, a silylated *tert*-butyl-calix[8]arene coated 433 MHz SAW sensor was able to differentiate tetrachloroethylene from *n*-heptane by a factor of 15. Furthermore, it was also observed [63] that the SAW sensor response remains almost unchanged from 20% to 60% relative humidity. In terms of selectivity between aromatic analytes, a smaller size calixarene e.g., *tert*-butylcalix[4]arene can distinguish *o*-xylene from toluene whereas *tert*-butylcalix[6]arene exhibited similar sensor responses to these analytes [64]. The comparison between different sizes of calixarene structures suggests that geometrical adaptation of the target analyte is important for the stable host-guest complex formation.

### 3.2. Metal Oxide Nanofilms and Composites

**Figure 6:**
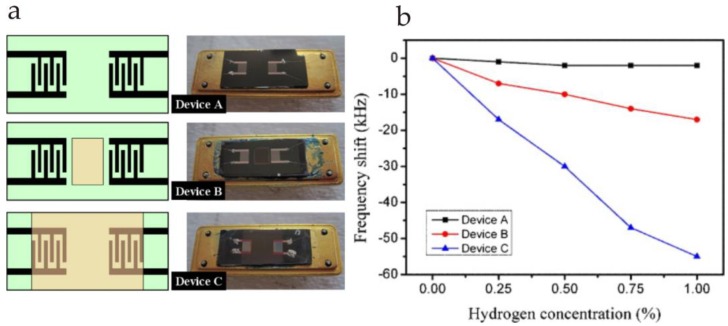
(**a**) SAW devices **A** (uncoated), **B** (conventional Pt/ZnO layer) having a chemical recognition layer between two sets of IDTs and device **C** (layered Pt/ZnO) which has a sensor layer extended on IDTs; (**b**) Sensor response of three devices **A**, **B** and **C** measured at room temperature and relative humidity of 30% for H_2_ gas (0–1%). Adapted with permission from [76], copyright (2012) Elsevier.

Metal oxide nanostructured layers are some of the most extensively studied gas sensor materials [65,66,67,68,69] because of their outstanding electrical properties. Along with SAW, there are growing numbers of research articles related to VOC detection for diverse applications. Small particle size with enhanced surface area make them highly sensitive to low analyte concentrations. A diverse range of metal oxide nanostructures are used as SAW interfaces, however, zinc oxide (ZnO) and tungsten oxide (WO_3_) are among the most frequently used nanomaterials [70,71]. For instance, Raj and coworkers [72] used ZnO with a one port 433.92 MHz SAW device for ammonia detection. The authors explained the differential frequency shift (∆*f*) on the basis of mass, electrical and elastic loadings. They proposed that elastic loading is dominant for ammonia gas sensing whereas mass effects are significant for liquid ammonia detection. In a later report [73], the authors compared the sensing properties of ZnO with those of other metal oxides, i.e., SnO_2_, TeO_2_ and TiO_2_, having the same layer thickness of 40 nm. They explained that the adsorption of ammonia and water molecules is a single step process. It is important to mention here that when ammonia and water are tested separately, elastic effects dominate, whereas when ammonia and water are together, mass loading effects take control. Furthermore, the experimental SAW sensor data shows good agreement with the mathematical equations, thus, mass and elastic loading effects could be explained. The comparative study has shown that ZnO is more sensitive for ammonia vapors than other metal oxide structures. They further investigated the different parameters that influence the liquid ammonia sensing mechanism of ZnO-coated SAW devices [74]. In another study [75], nanocrystalline ZnO thin films were coated on two-port SAW devices for humidity sensing. The effect of ZnO layer height on sensor response was also studied, which suggested that a 10 times coating (layer height of 300 nm) of ZnO film is more responsive than a six times coating (layer height of 180 nm). Phan and Chung [76] used ZnO nanoparticles fabricated with a Pt catalyst for H_2_ sensing using a two port AlN/Si SAW device. The authors measured the sensor response of three different SAW devices, i.e., an un-coated (device A), a conventional Pt/ZnO film (device B) and a Pt/ZnO layered surface (device C), as shown in Figure 6. They had found that the layered Pt/ZnO surface is more responsive than conventional sensing films because of the larger surface area of the layer and because it also covered the IDT electrodes. Moreover, the authors concluded that annealing treatment at 500 °C has a considerable influence on crystalline properties of ZnO, which ultimately leads to an enhanced sensor response for H_2_.

Metal oxide semiconductor-based gas sensors usually operate at elevated temperatures which require high power and special packaging, however, SAW devices are intended to work at room temperature. This urges the production of efficient metal oxide coatings that will be responsive to target vapors at lower temperature and therefore, can be combined with SAW. In this regard, there are number of research articles where a combination of two metal nanostructures is used to develop sensor coatings that are effective at low temperature. For example, tungsten trioxide (WO_3_) doped with Au nanoparticles has been used [77] for NO_2_ detection at low temperature. A recent study [78] has shown that Au nanostructures deposited on Ni IDTs can be used for vapor phase detection of mercury down to 1.3 ppb at 35 °C, which could be suitable for industrial applications. Jakubik and coworkers [79,80] used bilayers of WO_3_ and Pd films for hydrogen gas sensing. The purpose of combining two layers is to shift the *working point* to a high sensitivity region based on acousto-electric interactions. Thus, a small change in conductivity could perturb the SAW velocity which leads to larger frequency shifts. The porosity of metal oxide nanofilms [81] had a substantial impact on sensitivity, response and recovery times. Varghese and coworkers reported nanoporous alumina as a SAW interface for the detection of ammonia gas. The pore size of alumina was 43 nm whereas the layer height was 500 nm. The authors showed a fully reversible sensor signal for ammonia with a response time of 30–40 s and a recovery time of 60–80 s. Wen et al. [82] developed microporous WO_3_ films for NO_2_ sensing and observed that with pore sizes larger than 100 μm there was no appreciable response, but with a pore size of less than 1 μm, the recovery time becomes too long. The authors suggested that 50 μm WO_3_ films showed a good compromise between sensitivity and response time.

The combination of metal oxide nanoparticles with polymeric layers [83,84] was also tested for achieving room temperature vapor sensing. Sadek and coworkers [85] combined In_2_O_3_ (a well known gas sensing material) with polyaniline (a conducting polymer) for the detection of H_2_, CO and NO_X_. The developed SAW sensor showed good response, however, its selectivity and long term stability require further improvement. Dewan et al. [86] developed TeO_2_ thin films for NO_X_ sensing as well as temperature compensation layers. The authors found that optimal layer thickness of TeO_2_ results in enhanced sensitivity for NO_X_ and also reduced frequency shifts due to temperature variations.

### 3.3. Carbon-Based Nanomaterials

Carbon-based nanostructures such as carbon nanotubes [87,88,89], graphene sheets [90,91,92] and more recently diamond nanoparticles [93,94] are frequently used as gas sensor coatings. Their tunable chemical functionality, high surface to volume ratio, exceptional electrical features and enhanced thermal stability has made them popular in the chemical sensing domain. Unlike their inorganic competitors, i.e., semiconducting metal oxide layers, they can be used for room temperature sensing. The electronic properties of CNTs/graphene are changed when they interact with gas molecules depending upon their electron donor or acceptor nature. The charge transfer between adsorbed gas molecules and CNTs/graphene leads to change in electrical conductivity. To improve the recognition performance of CNTs, they are often dispersed in different polymeric matrices or combined with other metal oxide nanostructures. This also allows their suitable integration with transducers. There is a growing number of reports where carbon nanostructure-based coatings are used as SAW interfaces for vapor phase sensing.

Penza and coworkers have made significant contributions [95,96,97,98] in developing CNTs and their composites as SAW interfacial coatings for room temperature sensing of various VOCs. They studied the potential of single walled carbon nanotubes (SWCNTs) and multiwalled carbon nanotubes (MWCNTs) for vapor sensing. They proposed that mass loading of analyte molecules affects the wave propagation velocity, which results in resonant frequency shifts. The authors found that CNT-coated SAW devices result in much higher frequency shifts to VOCs compared to uncoated device. The selectivity was achieved by dispersing CNTs in solvents whose vapors were analyzed. Both SWCNTs and MWCNTs were dispersed in ethanol, respectively, showing higher sensor responses to ethanol vapors compared to ethyl acetate and toluene. The same was true when SWCNTs and MWCNTs were respectively dispersed in toluene. Sensitivity, absolute sensitivity and limit of detection are shown for both SWCNTs and MWCNTs in Table 2. The authors suggested that dispersion solvents are chemisorbed on CNTs surface and during sensing these solvent vapors are preferentially adsorbed. Thus, optimized matching of physiochemical properties of sensor materials to the respective analytes is performed. It was shown that SWCNTs are more sensitive and selective than MWCNTs. In case of using CNT composites with other materials, it is imperative to optimize the composition for high sensitivity. In another study [99], the authors explained that interaction between free charge carriers of nanocomposites and electrical field of SAW wave propagation leads to improved acousto-electric coupling, thus enhancing sensitivity.

**Figure 7:**
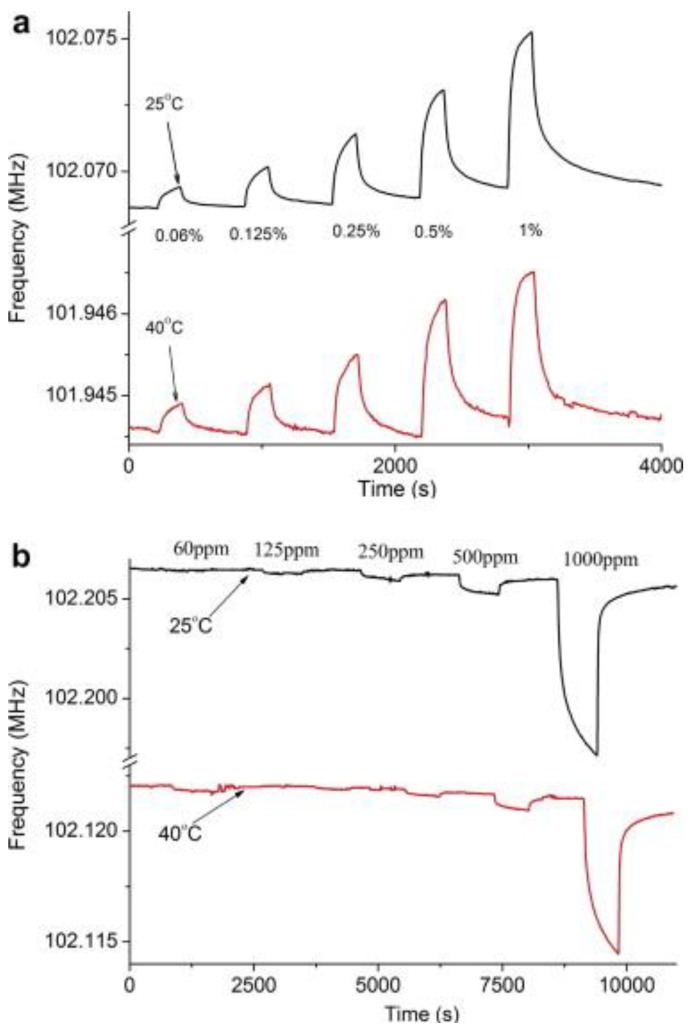
(**a**) Sensor response of graphene nanosheets integrated with LiTaO_3_ SAW device on H_2_ exposure showing an increase in frequency (**b**) Sensor response for CO showing drop in frequency, the measurements for both gases were carried out at 25 °C and 40 °C respectively. Adapted with permission from [105], copyright (2008) Elsevier.

Graphene-based gas sensors [100,101,102,103] are extensively studied by various research groups. The principle detection mechanism is based on conductivity shifts due to adsorbed gas molecules which change the electron density. SAW sensors are mainly gravimetric (mass sensitive) transducers however, the change in surface conductivity of these devices leads to altered wave propagation velocity. Thus, they can be used as acousto-electric transducers [104], depending upon their interfacial coatings. Arsat et al. [105] used reduced graphene oxide nanosheets for H_2_ and CO detection at two different temperatures i.e., 25 °C and 40 °C. Both these gases are reducing agents however, upon exposure to the SAW sensor, H_2_ resulted in an increase in the resonance frequency whereas CO showed a decrease in frequency. Figure 7a shows the increase in frequency on H_2_ exposure while Figure 7b represents the drop in frequency for CO. The authors explained that H_2_ is 14 times lighter in molecular weight than CO and this leads to conductivity changes in graphene sheets whereas for CO mass loading is the dominant factor that leads to a drop in frequency. The adsorption of H_2_ on graphene sheets leads to a drop in conductivity that would increase the velocity of acoustic waves and thus, an increase in frequency was observed. 

Furthermore, the authors showed that the developed sensor coatings are more responsive at 25 °C than 40 °C, which is ideally suited for room temperature sensing. Xuan et al. [106] used graphene oxide as a SAW interface for humidity sensing from 0.5% to 85%. They showed that thin graphene oxide layers, i.e., 70–90 nm, offer fast response times, i.e., <1 s, whereas thick layers, i.e., 200–300 nm, showed better sensitivity.

### 3.4. Functional Polymeric Layers

SAW sensors having polymer interfaces [107,108,109,110,111] have been extensively studied for a broad spectrum of applications. The structural and chemical diversity, straightforward integration with transducers, controlled layer heights and robust character in complex environments make polymer receptors a promising choice. There is a wide range of polymer-based receptor coatings that have been used as SAW interfaces for sensing both in gases and liquid phases. This includes pristine polymers [112,113], biomimetic polymers, i.e., molecular imprinted polymers [114], hyperbranched polymer coatings [115,116], conducting polymers [117,118], polymer composites [119,120] with nanomaterials and others. The recognition features of polymer layers can be tailored in different ways depending upon the size, shape and electronic interactions with analytes.

Polymer-based receptor coatings for SAW devices have been extensively studied by various research groups. The main objective of using pristine polymers as coatings is their inherent ability to respond specifically to a certain class of target analytes. For example, polymers having acidic functionality [121] e.g., can be used to detect basic analytes such as dimethylmethylphosphonate (DMMP) vapors [122]. In case of developing a sensor array, i.e., e-noses for analyzing complex vapor mixtures, a set of different polymers with tunable functionality can be used which makes them interact uniquely with each analyte. For data analysis, mathematical algorithms [123,124] such as principal component analysis [125], cluster analysis [126], artificial neural networks [127] and probabilistic neuronal network [128] have been used for pattern recognition. SAW e-nose sensor [129] arrays having polymer interfaces [130,131] are frequently used for analyzing vapors from different edibles including wine, vegetable oil and others. The developed sensor systems can be applied for classification as well as to detect adulteration in food samples.

**Figure 8:**
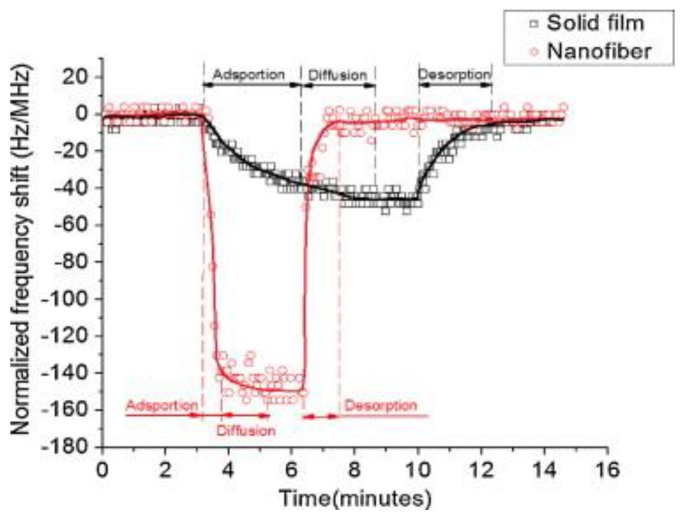
Comparison of the sensor responses of polyethylene oxide processed in two different forms, i.e., a *solid film* and *nanofibers*, explaining the adsorption, diffusion and desorption processes. It is obvious that polyethylene oxide nanofibers offer high sensitivity and faster kinetics. Adapted with permission from [132], copyright (2011) Elsevier.

Liu et al. developed polyethylene oxide nanofibers film as SAW interfacial coatings for sensing of hydrogen peroxide vapors [132]. The authors used an electrospinning process to produce polyethylene oxide fibers having 100–300 nm diameter and 8.1 μm thickness. From Figure 8, it can be seen that polyethylene oxide nanofibers offer faster kinetics of adsorption, diffusion and desorption as compared to solid film. Moreover, the sensor response of nanofibers is much higher than that of solid films. Figure 8 excellently explains the advantage of using nanofibers film over solid film for sensitive and rapid vapor phase sensing.

Molecular imprinted polymers (MIPs) are regarded as biomimetic receptors [133,134] as they follow typical antibody-antigen interactions. Here, the target to be analyzed is introduced as a template in a pre-polymer mixture where after curing, polymer chains engulf the template structures. The removal of template molecules yields highly adapted interaction sites in a polymer network which offer matching geometrical as well as chemical fit to target analytes. High percentages of cross-linker in MIP synthesis ensure the stability of the developed cavities and also enhance the chemical and thermal stability of the polymer layers. MIP coatings [135] along with SAW devices have been explored for a diverse range of analytes such as toxic vapors, microorganisms, polycyclic aromatic and halogenated hydrocarbons. For instance, the detection of *o*-xylene by a MIP-coated layer on a 433 MHz SAW and a 10 MHz QCM was, compared [136]. The observed limit of detection (LOD) for the SAW sensor was 0.1 μL/L, while for quartz microbalance it was 4 μL/L, thus indicating an increased sensitivity by a factor of 40. In another study [137], an imprinted polyurethane layer was combined with a 428 MHz SAW device for pyrene detection in water. The lowest detected concentration of pyrene was 3.5 ppb, which indicates high sensitivity of the MIP-based SAW sensor.

Wen et al. [138] had developed a MIP-based SAW sensor for detecting DMMP vapors using sarin acid as molecular template and *o*-phenylenediamine as functional monomer. The authors produced an exceptionally thin MIP film of a layer height of less than 10 nm and combined it with a 300 MHz SAW device having an insertion loss <12 db. The observed sensitivity for DMMP was about 96 Hz/mg/m^3^ whereas the calculated detection limit was 0.5 mg/m^3^. In another study [139], an increased sensitivity, i.e., 350 Hz for 0.1 mg/m^3^ DMMP was obtained using self-assembled imprinted calixarene layers. The most outstanding property of MIP recognition layers is their applicability for diverse targets, e.g., yeast cells. The detection of a single yeast cell [140] was carried out using a surface imprinted polyurethane layer combined with a 428 MHz shear transverse wave (STW) resonator having a LiTaO_3_ substrate. For working in liquids and complex mixtures, STW devices are capable of monitoring frequency shifts with reduced damping. Molecularly imprinted titania sol-gel coated STW resonators [45] have been successfully used for monitoring degradation products in oxidized engine oil. Highly stable and robust titania layer exhibited low noise, whereas imprinting effect favors the complete reversibility of the sensor signal. A sensitivity comparison was made using three STW resonators having different resonating frequencies but the same sensor coatings. The results showed that by increasing the frequency the sensor response increases in a parabolic manner for acoustic sensors. Polymer composites with nanomaterials such as metal nanoparticles and carbon nanotubes can lead to improved sensitivity. Nicolea et al. [141] combined polyethylenimine with SiO_2_/Si nanoparticles on a SAW surface for ethanol sensing. Sayago et al. [142] designed MWCNTs embedded in polyisobutylene as a SAW sensor layer for detecting octane and toluene at room temperature. The authors optimized the percentage of MWCNTs in the polymer matrix, i.e., to 2%, to obtain higher sensor signals.

### 3.5. Biological Receptors

The combination of biological receptors with SAW devices is a promising strategy for developing sensitive and selective biosensors [143] since the high operating frequency of a SAW ensures high frequency shifts whereas the biologically derived interfacial coating [144] would lead to selective binding. The foremost important aspect is label-free recognition which allows one to directly monitor layer-analyte interaction mechanisms as a function of frequency/phase shifts. Since the detection is made in liquid medium thus, for typical bio-sensing applications [145], Love Wave and (SH-SAW) devices are suitable choices. Bröker et al. [146] immobilized natural antibodies via a self-assembly process to confine gold nanospots on the SiO_2_ surface of a SAW chip. The shift in phase signal that corresponds to mass loadings is thus taken as the sensor response. They targeted circulating tumor cell (CTC) detection using anti-CD4 antibodies. The authors were able to detect human placental choriocarcinoma and lymphoblastic leukemia cancer cell lines. The comparison between *complete gold coverage* for antibody immobilization and the *modified gold nanostructure-* attached antibody receptors suggested that the latter strategy resulted in enhanced binding affinity for placental choriocarcinoma cell detection without disturbing the selectivity. The signal achieved by a *complete gold surface* at 1.6 × 10^6^ cells/mL concentration can be achieved by a *modified gold surface* at 80 times smaller concentration, i.e., 2 × 10^4^. Lange et al. [147] integrated monoclonal anti-urease with SH-SAW for observing urease binding. They first covered gold electrodes on a LiTaO_3_ substrate by a thin parylene layer and then used a dextran derivative, i.e., optoDex-A, to couple antibodies. The purpose of using parylene is to produce uniform films offering good adhesion while the dextran hydrogel is suitable in reducing unspecific binding interactions. The developed sensor showed high sensitivity and required only 60 nL cell volume for measurements, which is useful in bio-sensing where small samples are available.

**Figure 9:**
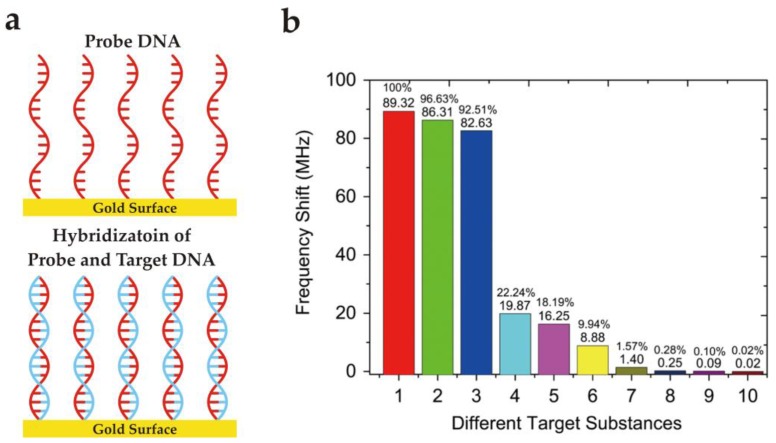
(**a**) Schematic representation of *probe* and *target* DNA hybridization at gold surface. (**b**) Comparison of frequency shifts for different samples. Adapted with permission from [148], copyright (2015) Elsevier. In this graph, **1:** Target DNA (1 μg/mL) having 15 bases; **2:** Non-target DNA (1 μg/mL) having 15 bases with one mismatch base sequence; **3:** Non-target DNA (1 μg/mL) having 15 bases with two mismatch base sequence; **4**: Non-target DNA (1 μg/mL) having only 4 bases; **5**: Non-target DNA (1 μg/mL) having only 3 bases; **6**: Target DNA (0.1 μg/mL) having 15 bases; **7**: Target DNA (0.01 μg/mL) having 15 bases; **8**: Target DNA (0.001 μg/mL) having 15 bases; **9**: BSA sample; **10**: PBS buffer.

Recently, Cai et al. [148] reported an exceptionally high resonating frequency SAW biosensor, i.e., 6.4 GHz device, for DNA sequencing and the detection of living cancer cells. Using such a high resonating frequency, a quality (Q)-factor of more than 4000 was achieved. The authors used a thiol- modified DNA probe to link it with the gold electrodes of a SAW delay line in a controlled environment while the uncovered gold surface was coated with bovine serum albumin (BSA). The developed sensor exhibited a linear response to target DNA sensing in the range of 1 μg/mL to 1 ng/mL, having a sensitivity of 6.7 × 10^−16^ g/cm^2^/Hz. This allows the detection of single hybridized DNA bases which is remarkable. Furthermore, the detection of single mouse mammary adenocarcinoma and fibroblast cells was also studied. Figure 9 shows the frequency shifts for different DNA samples including target and non-target substances. Additionally, the responses for BSA and buffer were also included. From Figure 9b **1** and **2**, it can be seen that DNA samples having only one mismatched base sequence in sample **2** can be differentiated by the designed setup. The sample **3** had two mismatched bases and thus showed even less response than **2**. Furthermore, DNA samples **4** and **5** having the same concentration but different number of bases give much lower frequency shifts due to the reduced masses. Samples **6**–**8** represent the sensor responses of the same DNA with varying concentrations, i.e., starting from 0.1 μg/mL to 0.001 μg/mL. Finally, **9** and **10** correspond to BSA and buffer showing negligible responses. The development of such a highly sensitive (capable of atomic resolution according to the mathematical calculations) selective and label-free biosensor exhibits its potential for diagnostic applications.

Zhang et al. [149] used a DNA aptamer as bio-recognition layer combined with a microfluidic Love Wave device for prostate specific antigen (PSA) detection. They used a LiTaO_3_ substrate having s SiO_2_ guiding layer whereas subsequently Cr/Au layers were also developed to immobilize the aptamer. The authors used a two channel device that has frequencies of 197 MHz and 198 MHz. The detection channel was treated with PSA solution whereas 1% BSA was injected into the reference channel. The purpose of using s reference channel was to study the temperature-frequency variations. The developed sensor showed a good linear response, i.e., in the range of 10–1000 ng/mL, for PSA detection.

## 4. Challenges and Opportunities

From the examples described above it is evident that SAW sensors have emerged as promising smart devices that can be used in diverse fields. Starting from defense applications, they can be applied to environmental monitoring of toxic and hazardous vapors, food analysis and control, engine oil ageing, microorganism and more recently cancer cell detection. However, there are certain issues related to transducer design and recognition layers as well which need to be addressed to make them viable in the commercial sensor field.

### 4.1. Device-Related Issues

The number of gaseous/vapor phase sensing applications is much larger than that of liquid phase ones, which is due to some inherent limitations of classical SAW devices. This problem has been solved by using SH-SAW and STW resonators and additionally, the use of guiding layer of sub-micrometer thickness in Love Wave devices is suited for liquid phase sensing. Nonetheless, these devices have not been extensively studied with microfluidic packages. Integrating SAW with microfluidic [150] and lab-on-chip (LOC) systems could realize their applications for precise and accurate liquid phase measurements, especially for biosensing [151]. The combination of SH-SAW and polydimethylsiloxane (PDMS) microfluidic systems for bioanalytes has been reported [152,153]. However, the flow rate fluctuations, mixing and pumping related problems have to be solved for optimized performance. Certain variables, including the temperature and relative humidity during gas phase sensing could lead to unwanted frequency shifts which need to be separated. The humidity effects can be reduced by using hydrophobic layers or by employing a separate SAW humidity sensor to continuously monitor the humidity during measurement and subtracting its effect to have a net response. In order to avoid frequency shifts due to temperature fluctuations, dual channel SAW delay lines having a temperature compensation element can be used. Furthermore, this issue can also be addressed by selecting suitable piezoelectric materials having small TCF values.

### 4.2. Interfacial Receptors

We have listed a variety of different coating materials used as SAW recognition layers. These receptor materials vary in their synthesis, processability, integration with the transducer surface and their recognition features, including sensitivity and selectivity. Additionally, most of the recognition coatings are only used for vapor phase applications. It is also imperative to mention here that analyte-layer interactions need to be highly specific to avoid any false frequency shifts due to unspecific binding. The advantage of using SAW is their label-free gravimetric sensing principle, since every analyte has a mass and its undesired interaction with the receptor coating could lead to frequency shifts, therefore, highly specific recognition layers are required for accurate analysis.

Supramolecular structures mainly recognize analyte vapors based on complementary geometrical fitting and favorable host-guest interactions. They overcome relative humidity problems by functionalizing the host structure with hydrophobic groups and furthermore, can be easily fabricated with transducer for having smooth layers. However, these receptors require extensive laboratory preparation to have the desired functionality for selective interaction with target vapors. Metal oxide nanomaterials are well known gas sensing materials, but they are used at high temperatures which are not suitable for routine SAW operation. Carbon-based nanostructures including CNTs and graphene oxide offer exceptionally sensitivity due to their high surface area, outstanding electrical and mechanical properties. The selectivity of such coatings can be improved by homogenously processing them in composite/hybrid forms e.g., with functional polymers like CNTs dispersed in polyisobutylene [142] which have shown good sensitivity and selectivity. Furthermore, the integration of such nanocomposites with the SAW surface is easy compared to CNTs alone.

Functional polymer coatings offer the advantage of chemical diversity so that receptor layer can be functionalized according to the target analyte. This indeed is promising for achieving high selectivity and building sensor arrays for multiplex analysis. While working in a complex environment and dealing with variety of analytes, it is important that each receptor responds uniquely to every analyte in a mixture. Functional polymeric receptors such as imprinted polymers offer high selectivity moreover. They can also be coated as homogenous thin films that lead to fast response and complete recovery of the signal. For improved sensitivity, polymeric layers can be processed in nanofibers [132] which improve the sensitivity compared to solid thin films.

### 4.3. Emerging Trends and Opportunities

For a long time, SAW devices remained exclusively in the domain of gas sensors but now we have seen that sensing applications in liquid phases, including viscoelastic fluids [154,155], are being reported more frequently using SH-SAW and Love Wave devices. This would certainly extend the scope of surface acoustic devices to other fields, especially in biosensing. For example, the combination of biologically-derived receptors such as natural antibodies, DNA, or aptamers with these devices could lead to the development of reliable biosensors. The inherent selectivity of these materials ensures specific analyte binding. The high operating frequency of acoustic transducers would result in high sensitivity capable of detecting ultra-low concentrations. For example, this is extremely important to detect low concentrations of protein biomarkers in blood serum or urine samples for early stage diagnostics. In a recent study [156] a SAW sensor was developed for differentiating female *Aedes* mosquito from male species based on their wing beat frequencies which could be useful in indentifying clean environments free from dengue infections. Being of small size, miniaturization is straightforward and it can be integrated with data processing unit. SAW biosensors could be adopted in point of care (POC) testing for diagnostics of different diseases. For examples, cancer cell detection using microfluidic Love Wave devices having aptamer interfaces is a recent example of such a system. Moreover, the use of high resonance frequencies in biosensors allows mathematical determination of atomic scale resolution. Even apart from liquid phase operation, SAW sensors combined with synthetic polymers were used to detect lung cancer non-invasively [157]. Human breath contains a number of VOCs, including clinically significant biomarkers of lung cancer. These markers can be detected using SAW sensor arrays [158] having polymer receptors and the data can be interpreted by a imaging recognition method combined with artificial neural network technology. However, in all biosensing applications of SAW devices, sample pre-treatment or pre-concentration is an important step to have more precise results.

**Figure 10:**
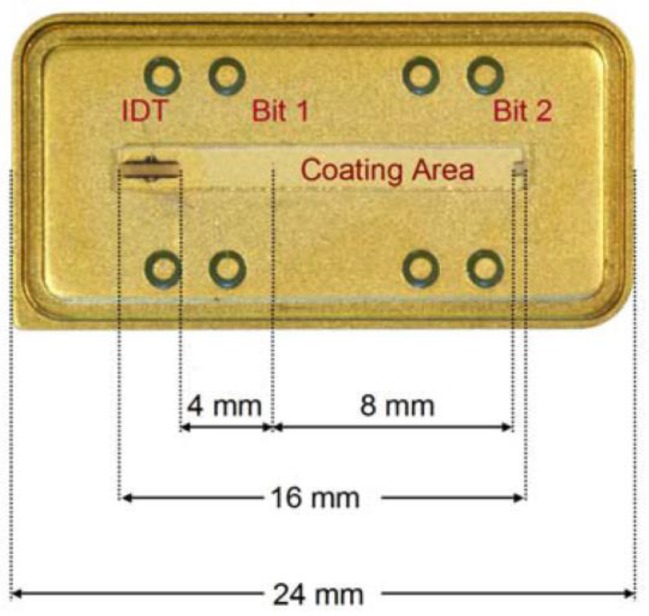
A typical RFID-tag coated with polyvinyl alcohol for humidity sensing. The figure shows the design details of the device, including the dimensions of the IDT, coating area and distances between IDT, Bit 1 and Bit 2 (reflections). Reproduced from [160], copyright Molecular Diversity Preservation International (MDPI).

SAW devices are highly suitable for wireless communications and thus, combined with molecular recognition layers, they can be designed for remote sensing applications [159]. Such an attempt [160] has been made where a chemical sensitive layer, e.g., polyvinyl alcohol, is fabricated with RFID-tags for relative humidity sensing applications. Figure 10 shows the design details of such a device having a special coating area. The combination of chemical recognition layers with RFID devices could lead to the development of smart remote chemical sensors. However, real time applications demand optimized transducer design and highly specific molecular recognition coatings.

In general, the biosensing aspect [161,162,163,164,165,166] of SH-SAW/Love Wave devices remained unexplored for a long time, but during the last few years some promising results have been published which indicates the potential of these devices in biosensors [167]. SAW-based commercial sensing systems are already in the market for the detection of explosives and narcotics that can be used for screening as well as bulk analysis. The combination of gas chromatography with SAW sensors is desirable where a prior separation of a complex mixture is carried out by a gas chromatography column followed by SAW sensing. zNose sensor technology [168] developed such a sensor system for real time analysis of chemical vapors. Such devices can be used for diverse needs such as chemical security, agriculture and food safety. These examples suggest that SAW devices for VOCs sensing are making their way from laboratory handheld devices [169] to mature sensing tools [170]. In the sensor market however, the biosensing aspect needs further efforts and research to make them an established technology.

## 5. Summary and Outlook

In this review article, we have explained the combination of SAW devices with a range of diverse recognition layers for chemical sensor applications. The device part covers the basic transduction mechanism, selection of substrate material, design and development details, operation in gas and liquids, and temperature frequency sensitivity relationships. The recognition layers, starting from supramolecular structures to small nanoparticles/nanotubes/nanofibers, functional polymer and their hybrids, and more recently biologically derived receptors are included. These recognition layers are discussed in view of their responsive character for a particular class of analytes with selected examples from the literature. We have seen that SAW sensors are more extensively reported for gas phase analysis however, with certain modifications of the basic SAW design, liquid phase sensing is also possible. The foremost advantage of using acoustic devices is their label-free transduction principle. However, at the same time every analyte has a mass and this could lead to unwanted frequency shifts. Therefore, the use of highly selective recognition layers including both natural as well as synthetic receptors could result into specific binding responses. Portable SAW sensors for vapor phase applications are already in the market but liquid phase sensing, particularly for the detection of bio-analytes, has yet to reach an established point. Future research should focus on the development of high resonating frequency SAW devices capable of liquid phase operation and combined with selective and robust recognition layers for improved performance in bio-sensing and other domains.

## Figures and Tables

**Table 1:** Comparison of different piezoelectric materials in terms of their dielectric constants, TCF, SAW propagation velocity and general comments for SAW manufacturing.

Piezoelectric Material	Dielectric Constant	TCF ^a^ (ppm/°C)	Max. Working ^b^ Temperature	Velocity (m/s)	Comments
Quartz (ST-X)	3.8	0	573 °C	3159	Common and inexpensive, Stable against temperature, Not suitable in aqueous phase.
LiTaO_3_ (X-112° Y)	43	18	≈300 °C	3300	High frequency SAW devices, Suitable for liquids phase operation, Temperature drifts
LiTaO_3_ (36° Y-X)		32		4160	
LiNbO_3_ (128° Y-X)	85.2	75	≈300 °C	3979	High frequency SAW devices, Suitable for SH-SAW liquid phase, Larger TCF values
LiNbO_3_ (64° Y-X)		80		4742	
LiNbO_3_ (Y-Z)		94		3488	
AlN	8.5	19	≥1000 °C	5700	High frequency SAW devices and used in form of thin layer on other substrates, High thermal stability
La_3_Ga_5_SiO_14_	18.23	≈0	1470 °C	2734	Enhanced piezoelectricity, High thermal stability Low insertion loss

^a^ TCF values for quartz, lithum tantalite (LiTaO_3_) and lithium niobate (LiNbO_3_) were taken from [38]. ^b^ The maximum working temperature data is derived from [39]. The data about aluminum nitride AlN is reported from [40] and for langasite (La_3_Ga_5_SiO_14_) it is taken from [41].

**Table 2:** Comparison of sensor responses of 433.92 MHz SAW coated with SWCNTs and MWCNTs dispersed separately in ethanol and toluene. Adapted with permission from [97], copyright (2005) Elsevier.

**Type of VOC**	**SWCNTs Dispersed into Ethanol**				**MWCNTs Dispersed into Ethanol**			
	**Sensitivity *(*∆*f/f)/c*** **(ppm/ppm)**	**Sensitivity** **∆*f/c*** **(kHz/ppm)**	**LOD (ppm)**	**Absolute Sensitivity** **∆*f/c/*∆*f_w_*** **(Hz/ppm/kHz)**	**Sensitivity *(*∆*f/f)/c*** **(ppm/ppm)**	**Sensitivity** **∆*f/c*** **(kHz/ppm)**	**LOD (ppm)**	**Absolute Sensitivity** **∆*f/c/*∆*f_w_*** **(Hz/ppm/kHz)**
**Ethanol**	15.88	6.89	1.3	34.45	4.13	1.79	5.0	8.95
**Ethyl Acetate**	7.67	3.32	2.7	16.6	2.09	0.90	10.0	4.50
**Toluene**	7.32	3.17	2.8	15.85	2.33	1.01	9.0	5.05
	**SWCNTs Dispersed into Toluene**				**MWCNTs Dispersed into Toluene**			
	**Sensitivity *(*∆*f/f)/c*** **(ppm/ppm)**	**Sensitivity** **∆*f/c*** **(kHz/ppm)**	**LOD (ppm)**	**Absolute Sensitivity** **∆*f/c/*∆*f_w_*** **(Hz/ppm/kHz)**	**Sensitivity *(*∆*f/f)/c*** **(ppm/ppm)**	**Sensitivity** **∆*f/c*** **(kHz/ppm)**	**LOD (ppm)**	**Absolute Sensitivity** **∆*f/c/*∆*f_w_*** **(Hz/ppm/kHz)**
**Ethanol**	14.62	6.34	1.4	31.70	2.02	0.87	10.2	4.35
**Ethyl Acetate**	12.58	5.45	1.6	27.25	3.99	1.73	5.2	8.65
**Toluene**	17.22	7.47	1.2	37.35	4.67	2.02	4.4	10.10

Measurements were conducted at room temperature 20°C, relative humidity 15%, exposure time of 10 min, noise level 3 kHz at S/N of 3. *(*∆*f/f)* represent fractional frequency shift, ∆*f* frequency shift, c concentration of vapors, *(*∆*f_w_)* frequency shift due to coating of CNTs.
